# Management of Lateral Thigh Pain following Cephalomedullary Nail: A Technical Note

**DOI:** 10.51894/001c.12931

**Published:** 2020-06-08

**Authors:** Michael Rosen, Connor Kasik, Michael Swords

**Affiliations:** 1 Michigan State University College of Osteopathic Medicine; 2 Michigan Orthopedic Center

**Keywords:** hardware irritation, lateral hip pain, complication, cephalomedullary nail

## Abstract

Peritrochanteric hip fractures are most commonly treated with proximal femoral fixation devices, such as a cephalomedullary nail or sliding hip screw. As usage rates increase for these fixation devices, complications from their insertion are becoming more prevalent. Lateral hip pain from proximal locking device insertion and prominence continues to be one of the most frequent complaints regarding hardware irritation following this surgical procedure. Conservative treatment options for this complaint include local corticosteroid injection and physical therapy, although once these treatments have been exhausted, surgical intervention may be recommended. This has generally been managed previously with implant removal, although studies have shown associated femoral neck fractures after removal even with the prescribed protected postoperative weight bearing. Additionally, in certain situations (e.g., when the nail is placed for prophylactic treatment), its removal is contraindicated. The purpose of this manuscript is to describe an alternative treatment option that would limit morbidity, and the need for proximal locking device or implant removal by excising the portion of the iliotibial band causing hip irritation at the level of the proximal locking device, while leaving the retained implant in place. This surgical option would allow most patients to return to their pre-operative weight-bearing status immediately following surgery without the need for additional postoperative precautions.

## INTRODUCTION:

Usage of proximal femoral fixation devices, such as cephalomedullary nails (CMN) and sliding hip screws (SHS), for peritrochanteric femoral fractures continue to rise, increasing from a rate of 3% in 1999 to 67% in 2006.^1^ The additional prevalence of these implants leads to an increased incidence of the associated complications, with little attention often paid to persistent pain after successful fracture union.^2^ Over 40% of patients report pain after nail placement,^2^ one of the most common being lateral hip pain over the greater trochanter.^3,4^

CMN and SHS implants facilitate a dynamic compressive mechanism at the fracture site by allowing shortening (compression) at the fracture with weight bearing. Symptomatic lateral hip pain may develop as the fracture compresses on the proximal locking device (PLD), creating increased hardware prominence.

Options to address this evolving postoperative problem may include conservative treatment with local steroid injection at the site of maximal tenderness, physical therapy or invasive options including isolated PLD removal or complete hardware explantation. However, several reports can also be found in the literature describing associated femoral neck fractures following removal of hardware.^5–7^

In addition, after hardware removal, patients must maintain protected weight bearing postoperatively to minimize fracture risk, although no definitive time is agreed upon for return to full activity.^5–7^ In specific situations, implant removal may be contraindicated, such as when placed for impending fracture as prophylactic treatment.

## PROCEDURE DEVELOPMENT

The technique described in this report was developed by the authors to alleviate lateral hip pain from PLD irritation. Proper patient selection is key, as additional symptoms (e.g., thigh pain) are not addressed. Physical exam may be solely relied upon for identification, or diagnostic injection may also be used. Surgical intervention may be pursued once injection is proven to provide effective pain relief.

Our technique involves resection of hypertrophic bursa while creating a window in the tensor fasciae latae (TFL) and iliotibial band (ITB), thereby removing impinging tissues around the PLD. The advantage of this technique is that it avoids hardware removal and the associated fracture risk without the restrictions in immediate weight-bearing status. To our knowledge, this alternative technique has not been discussed elsewhere in the literature.

## PROCEDURE DESCRIPTION

**Figure 1.**
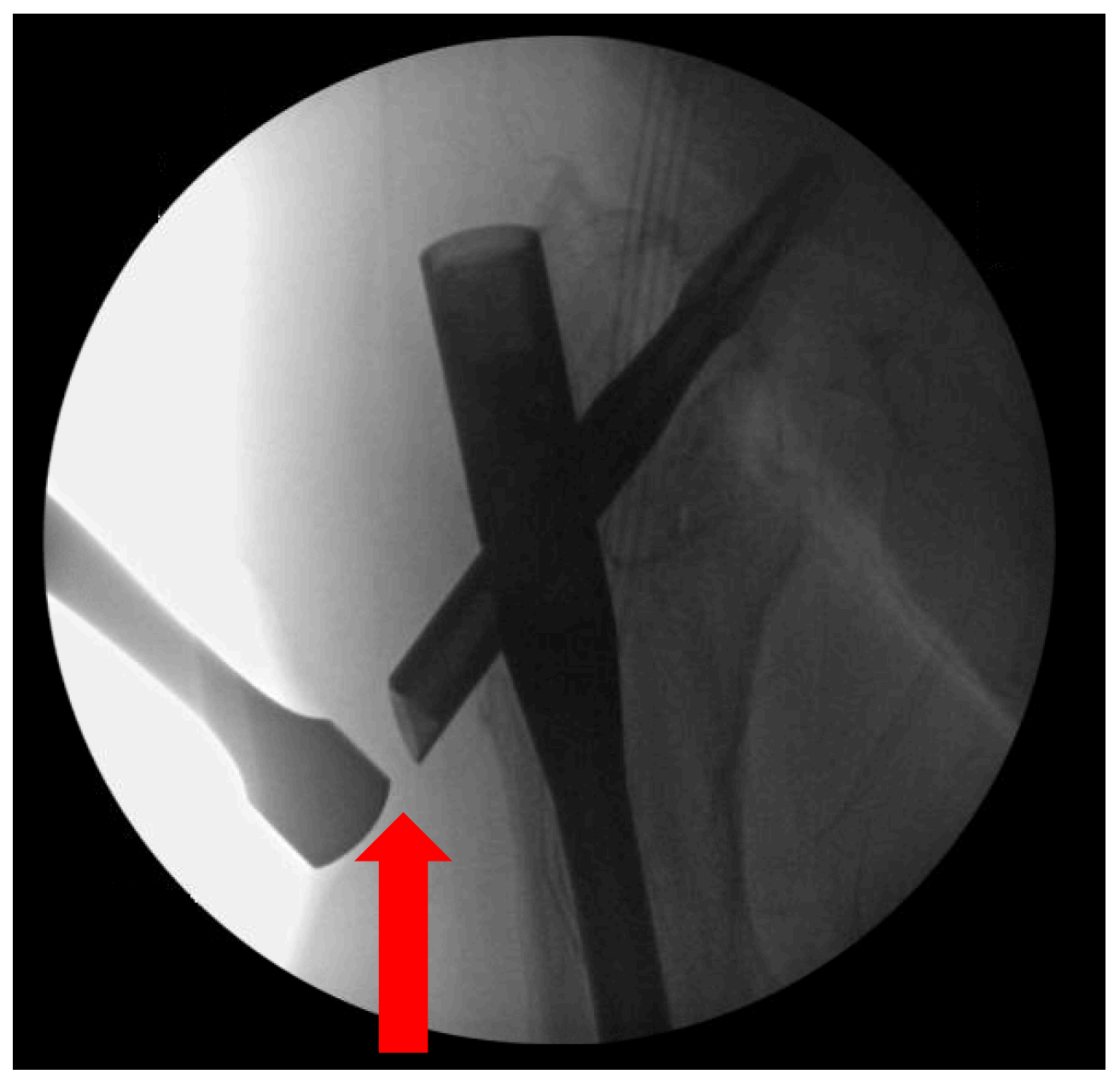
Fluoroscopic images used to localize incision site at prominence of proximal locking device (red arrow).

The patient is placed on a radiolucent Jackson flat top table with a beanbag to facilitate a “sloppy lateral” position. The entire operative extremity is prepped and draped independently of the well leg, allowing the surgeon to assess a full range of motion following TFL and ITB excision. Intra-operative fluoroscopy is used to localize the appropriate starting point for the incision at the lateral end of the PLD, while utilizing previous surgical scars. (Figure 1)

**Figure 2.**
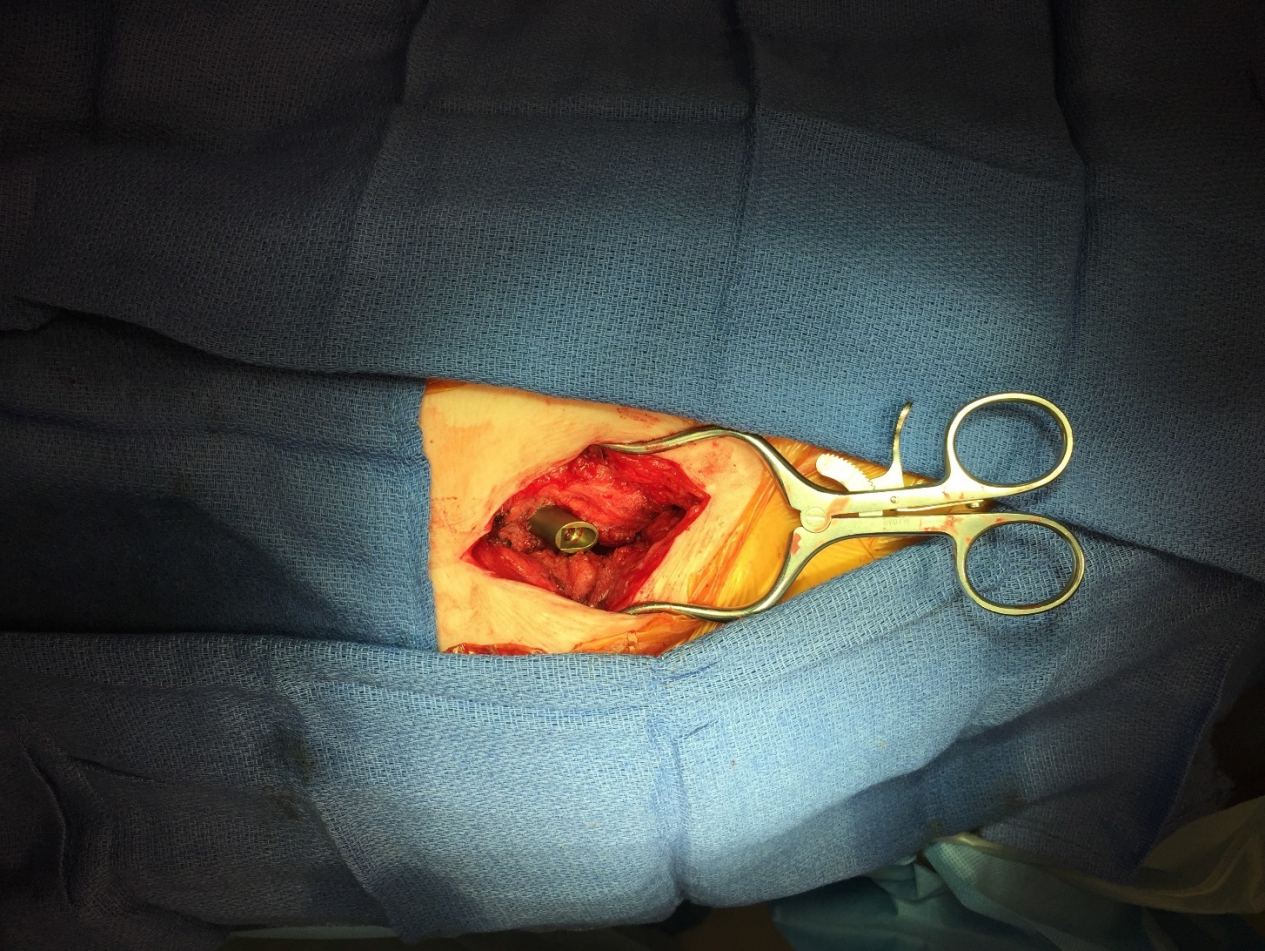
Initial exposure following elliptical excision of ITB and visualization of proximal locking device with surrounding fibrosis and hypertrophic bursal tissues.

**Figure 3.**
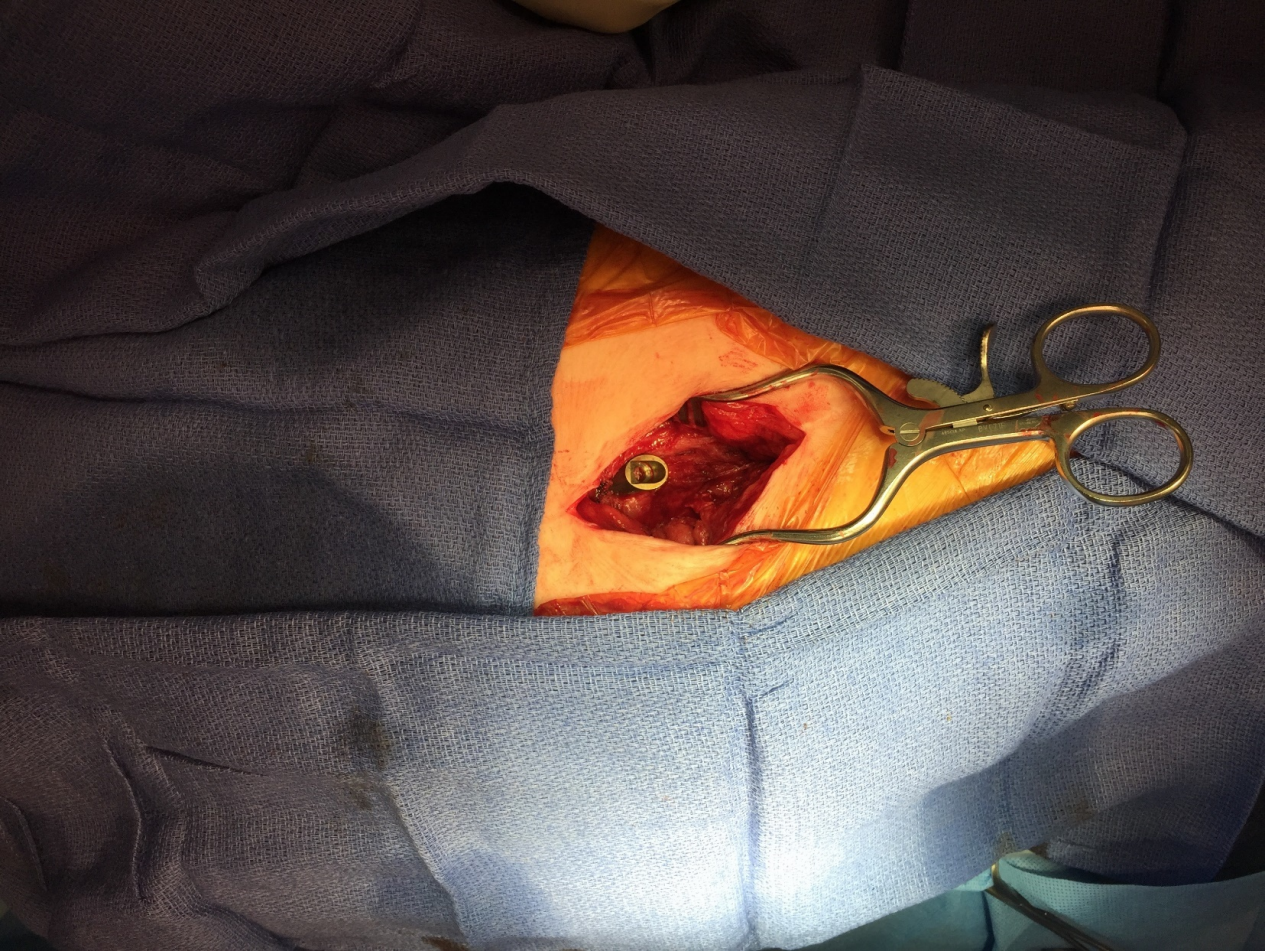
Following removal of fibrotic tissues.

A longitudinal incision is made with superficial dissection carried down to the ITB, using electrocautery for hemostasis. (Figure 2) The patient’s hip is then brought through a complete range of motion to visualize area of impingement on hypertrophic bursal tissue and ITB; these tissues are then elliptically resected. (Figure 3) No portion of the implant is removed.

**Figure 4.**
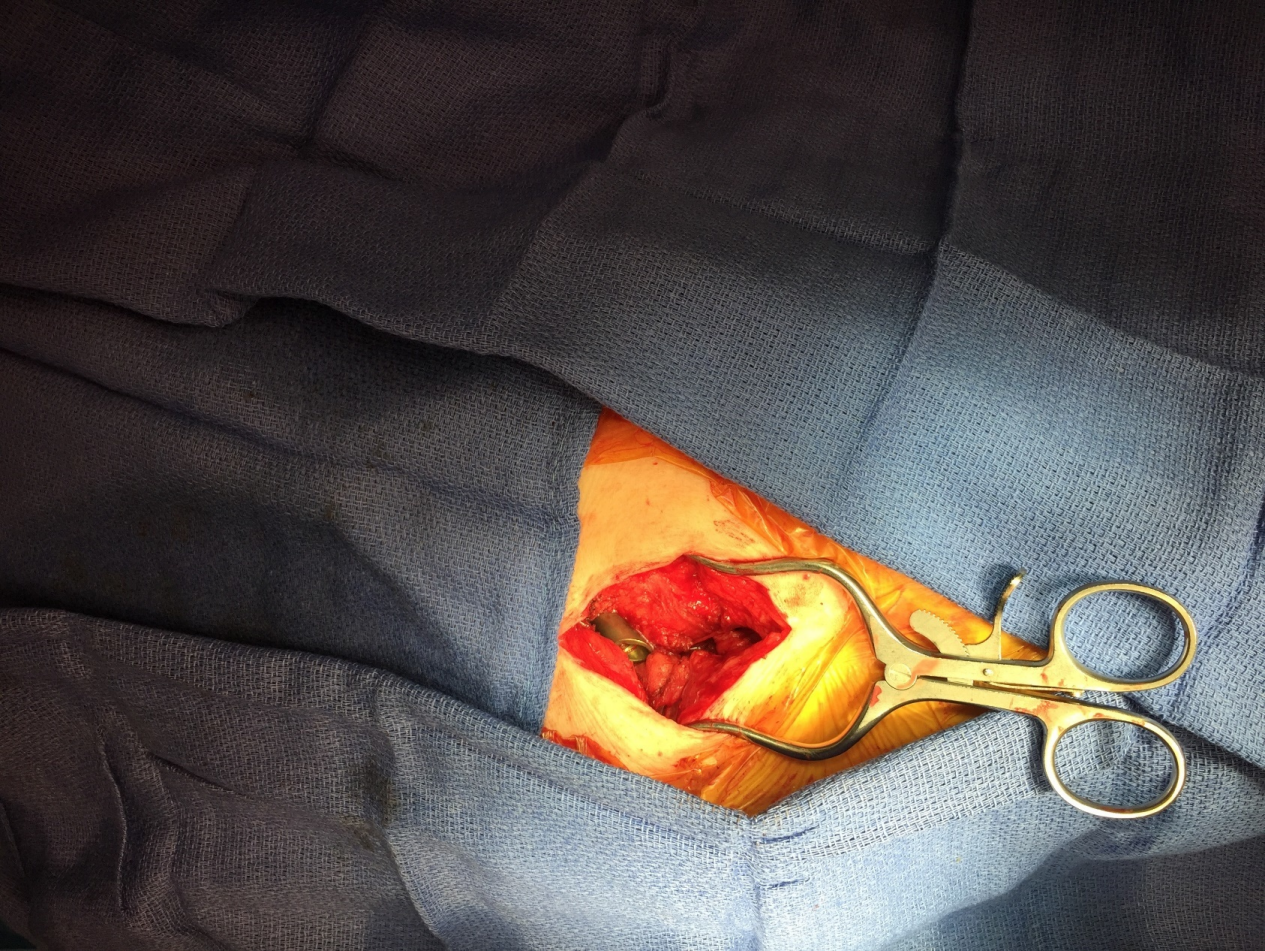
Verification of adequate fibrotic tissue excision while taking hip through range of motion, here the hip tolerates abduction and external rotation with no evidence of impingement.

**Figure 5.**
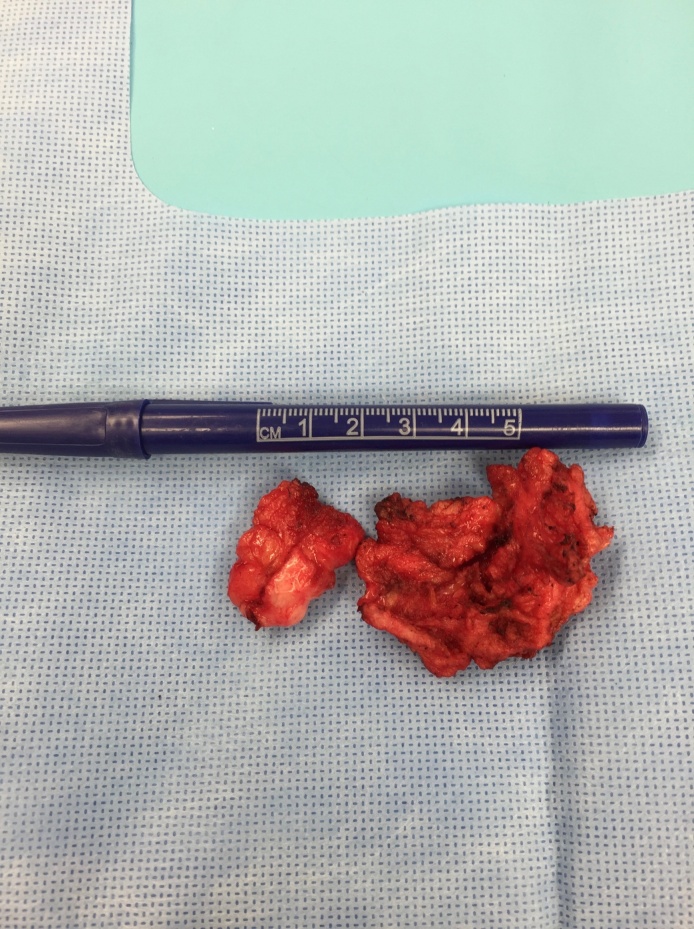
Fibrotic tissue following proximal hip excision.

Intra-operative reassessment of range of motion is performed in complete flexion-extension, internal-external rotation, and adduction-abduction to verify adequate excision of the impinging tissues. (Figures 4 and 5) The overlying skin incision is closed, leaving the ITB windowed and free of soft tissue impingement. Postoperatively, the patient is discharged the same day and allowed immediate full weight bearing to tolerance.

## DISCUSSION:

In the literature to date, persistent pain after radiographic union has been commonly treated with hardware removal; lateral hip pain being the most common complication after CMN.^3^ Hesse and Gachter examined a population of 498 patients and found implant-related complications in 42 patients (8%), 30 (71.4%) of which was trochanteric pain necessitating nail removal.^3^ Additional reported non-pain complications included delayed wound healing, cardiac or respiratory issues, decubitus ulcers or renal failure.^7^

Differentiating lateral hip pain from thigh pain is one key to determining appropriate treatment. In one study, Dodenhoff, et al. retrospectively reviewed the incidence and causes of persistent pain in the proximal thigh following hip fractures. Of 80 patients, 33 (41%) reported pain that was enough to interfere with their lifestyle or mobility, with 53 (66%) reporting pain in the trochanteric region. Meanwhile, 22 (27%) patients reported experiencing proximal thigh pain.^4^

In this same study, pain relief was found to be unpredictable following nail removal, and this group found no difference in pain with nail prominence above the tip of the greater trochanter.^4^ In view of these earlier numbers, our technique is an appropriate treatment option for most patients complaining of postoperative pain, after the correct etiology has been identified.

An accurate physical examination is necessary to differentiate PLD impingement from other common proximal femoral fixation device complications, such as thigh pain. Palpation at the PLD insertion site and limitation in hip range of motion in combination with the Ober test (i.e., procedure to test ITB tightness) may be used to differentiate the patient’s symptoms from nail related thigh pain.^7^ Steroid injection at the point of maximal tenderness may be used as a diagnostic test to alleviate symptoms while greater localizing the source of pain. A failure of symptomatic improvement after diagnostic injection indicates that additional etiologies should be addressed.^7^

Conservative treatment options (e.g., activity modification, physical therapy, and injections) for lateral hip pain in this setting are like the treatments for trochanteric bursitis and external snapping hip. If pain persists, surgical intervention is indicated and up to this point has included isolated PLD removal and proximal femoral fixation device explant.

In 2012, Shaer, et al. reported on a patient with pain at the PLD insertion site, positive Ober test and temporary relief after steroid injection.^7^ At 21 months, with persistent pain after conservative treatment, the patient elected to have the PLD removed while leaving the CMN intact. He was discharged home with partial weight-bearing status and at six weeks postoperatively, began progressing to weight bearing as tolerated.

One postoperative week later, this patient experienced an atraumatic displaced femoral neck fracture. To avoid this complication, his surgeons concluded to replace the hip screw with a shorter one that may sit in more flush manner with the lateral femoral cortex, or fill the bone void with injectable calcium phosphate cement.^7^

Removal of the PLD from the proximal femoral fixation is not without additional morbidity risks. In the case of a cephalomedullary nail, the PLD is secured with a set screw locking mechanism at the proximal aspect of the nail. After successful fracture union with an intramedullary device, bony overgrowth may develop at the nail insertion site.

If a surgeon wishes to remove the PLD to exchange it for a shorter implant to avoid hardware prominence, the nail insertion site must be debrided of bony overgrowth including interdigitation of bone with threads in the channel for the set screw in order to disengage the set screw from the PLD. This can create additional surgical difficulty and increase patient morbidity while also increasing procedure time.

Cement augmentation has demonstrated a significant increase (21%) in load to failure for femoral neck defects after screw removal, although this may produce additional morbidity in the setting of subsequent ipsilateral hip surgery, such as conversion to total hip arthroplasty.^8^

Kukla et al. further established the increased risk of postoperative fracture in a cadaveric model study (N = 37).^9^ They found that three (8%) of patients who underwent CMN removal sustained femoral neck fractures with full weight bearing postoperatively. Of those patients who wished to have their implant removed, nine (24%) were due to pain at the PLD site. Fracture force required was found to be significantly lower following CMN explant compared to SHS and the femoral excavation control.^9^ The difference between CMN and SHS may be attributed to the larger size diameter of the PLD for the CMN. These results demonstrate the importance of implant retention, especially in those with CMN.

When attempting to avoid associated femoral neck fractures, the presence of the implant itself may not be sufficient to prevent fracture. For example, Graziano, et al. reported on a patient with a previously healed IT fracture which had been treated with SHS.^10^ The patient went to develop a femoral neck fracture four years after hardware implantation.

Prophylactic CMN placement in the setting of impending fracture or metastatic disease is one scenario where removal of symptomatic hardware is not a viable treatment option.^11^ In this setting, our technique presents as a safer, and more practical alternative to PLD removal or cement augmentation.

If proximal femoral fixation device removal is performed, the surgeon must determine the appropriate weight-bearing status, as no consensus exists at this time.^12^ Our proposed technique avoids the limitations in postoperative active level as the hardware remains intact. More importantly, the possibility of postoperative femoral neck fracture is minimized.

To this point, the senior author (MS) has performed the described technique on a small sample of six patients over a five-year period who had complained of lateral hip pain after proximal femoral fixation, and early results are promising. In this sample, there have been no cases of recurrent pain, persistent pain, or femoral neck fracture, with complete symptom resolution. No patients sought additional treatment. Controlled outcome studies will be necessary to more fully evaluate this technique.

## CONCLUSION:

As CMN usage rates increase, surgeons need to become more aware of the possible associated postoperative complications. Lateral hip pain is one of the most frequent complaints regarding hardware irritation following this common surgical procedure. Conservative treatment options for this complaint include local corticosteroid injection and physical therapy. Once these treatments have been exhausted, surgical intervention may be recommended.

The surgical technique described in this paper may minimize patient morbidity and allows more rapid postoperative recovery than implant removal, which has previously been utilized. Surgeons should be aware of several treatment options when dealing with postoperative complications.

### Conflict of Interest

The authors declare no conflict of interest.

## References

[ref-24975] Anglen J.O., Weinstein J.N. (2008). Nail or Plate Fixation of Intertrochanteric Hip Fractures: Changing Pattern of Practice. J Bone Jt Surg Am.

[ref-24976] Bednar D.A., Ali P. (1993). Intramedullary nailing of femoral shaft fractures: Reoperation and return to work. Can J Surg J Can Chir.

[ref-24977] Hesse B., Gächter A. (2004). Complications following the treatment of trochanteric fractures with the gamma nail. Arch Orthop Trauma Surg.

[ref-24978] Dodenhoff R.M., Dainton J.N., Hutchins P.M. (1997). Proximal thigh pain after femoral nailing. Bone Jt. J.

[ref-24979] Buciuto Robert, Hammer Richard, Herder Anders (1997). Spontaneous subcapital femoral neck fracture after healed trochanteric fracture. Clinical Orthopaedics and Related Research.

[ref-24980] Mendez A.A., Joseph J., Kaufman E.E. (1993). Stress fractures of the femoral neck following hardware removal from healed intertrochanteric fractures. Orthopedics.

[ref-24981] Shaer J.A., Hileman B.M., Newcomer J.E.. (2012). Femoral Neck Fracture Following Hardware Removal. Orthopedics.

[ref-24982] Strauss E.J., Pahk B., Kummer F.J.. (2007). Calcium phosphate cement augmentation of the femoral neck defect created after dynamic hip screw removal. J. Orthop Trauma.

[ref-24983] Kukla C., Pichl W., Prokesch R.. (2001). Femoral neck fracture after removal of the standard gamma interlocking nail: A cadaveric study to determine factors influencing the biomechanical properties of the proximal femur. J Biomech.

[ref-24984] Graziano G.P., Heck D.A., Misamore G.W. (1988). Fracture of the femoral neck after internal fixation. J. Trauma.

[ref-24985] Mirels H. (1989). Metastatic disease in long bones: A proposed scoring system for diagnosing impending pathologic fracutres. Clin Orthop.

[ref-24986] Busam M.L., Esther R.J., Obremskey W.T. (2006). Hardware removal: Indications and expectations. J Am Acad Orthop Surg.

